# Scale-Adaptive High-Resolution Imaging Using a Rotating-Prism-Guided Variable-Boresight Camera

**DOI:** 10.3390/s25206313

**Published:** 2025-10-12

**Authors:** Zhaojun Deng, Anhu Li, Xin Zhao, Yonghao Lai, Jialiang Jin

**Affiliations:** 1 College of Surveying and Geo-Informatics, Tongji University, Shanghai 200092, China; 2 School of Mechanical Engineering, Tongji University, Shanghai 201804, China; 2310373@tongji.edu.cn (X.Z.); 2330459@tongji.edu.cn (Y.L.); 2330361@tongji.edu.cn (J.J.)

**Keywords:** large FOV, super-resolution images, variable-boresight camera, rotating prisms, distortion correction

## Abstract

Large-field-of-view (FOV) and high-resolution imaging have always been the goals pursued by imaging technology. A scale-adaptive high-resolution imaging architecture is established using a rotating-prism-embedded variable-boresight camera. By planning to prism motion, the multi-view images with rich information are combined to form a large-scale FOV image. The boresight is guided towards the region of interest (ROI) in the combined FOV to reconstruct super-resolution (SR) images with the desired information. A novel distortion correction method is proposed using virtual symmetrical prisms with rotation angles that are complementary. Based on light reverse tracing, the dispersion induced by monochromatic lights with different refractive indices can be eliminated by accurate pixel-level position compensation. For resolution enhancement, we provide a new scheme for SR imaging consisting of the residual removal network and information enhancement network by multi-view image fusion. The experiments show that the proposed architecture can achieve both large-FOV scene imaging for situational awareness and SR ROI display to acquire details, effectively perform distortion and dispersion correction, and alleviate the occlusion to a certain extent. It also provides higher image clarity compared to the traditional SR methods and overcomes the problem of balancing large-scale imaging and high-resolution imaging.

## 1. Introduction

Field of view (FOV) and resolution are key parameters to evaluate the performance of sensors [1]. High-resolution imaging with a large FOV has a wide range of applications in medical diagnosis, military reconnaissance, and deep space exploration [2,3]. In fact, wide-field high-resolution imaging is a challenge because these parameters are inherently contradictory in theory. The improvement in resolution mainly stems from two limitations [4]. Firstly, the size of the pixels used to construct the sensor cannot be infinitely small. In addition, the imaging resolution is limited by the optical diffraction limit. At present, super-resolution (SR) technology is regarded as an effective way to enforce the imaging resolution close to the diffraction limit [5].

SR imaging technology, restoring high-resolution images from low-resolution images through image processing methods, has received key research and extensive attention in recent years [6,7]. According to the number of original images, it mainly includes single-frame SR imaging methods and multi-frame SR imaging methods. The single-frame SR imaging technology achieves high-resolution information recovery by establishing a mapping relationship between low-resolution and SR images in advance [8]. Zhang proposed an SR imaging method based on a single image, which can learn local dictionaries and non-local similar structures from the input image to reconstruct high-resolution details [9]. BSRGAN, a generative adversarial network (GAN) for super-resolution (SR) imaging, was proposed by Zhang [10]. Their work enables the recovery of realistic textures from sampled images in public datasets. However, the above algorithms require prior knowledge to build mathematical models and have insufficient universality. SR technology based on multi-frame images can recover the perceived scene in high resolution by fusing multiple low-resolution images [11]. Compared with the super-resolution technology using a single image, it does not require prior information and has better adaptability and generalization ability. For this type of technology, sub-pixel imaging is the key to improving image resolution [12]. Therefore, a series of sub-pixel imaging methods have been proposed, such as multi-time/view imaging [13], optical scanning imaging [14], and camera array imaging [15]. In addition, microscopic imaging techniques are also used to improve image quality, such as compact lens-free microscopic imaging techniques [16]. Although the perceptual resolution has improved, the perceptual range is limited.

Flexible imaging systems consisting of a camera and mirrors, as a common wide-range imaging system, have received significant attention [17]. Typical systems include flow detection scanners [18], multi-mirror scanning systems [19], and micro-electro-driven variable boresight systems [20]. Regrettably, these large-scale perception systems based on the principle of optical reflection have limitations such as large physical size, sensitivity to errors in processing and assembly, and large moment of inertia. Notably, Carles et al. proposed array cameras with wedge prisms to expand the imaging FOV [21]. Compared with array cameras, it has certain advantages in terms of integration and economy. In fact, Risley prisms have been widely used in beam control due to their high pointing accuracy, compact configuration, and good dynamics [22]. In particular, the forward and inverse solutions of rotating Risley prisms have undergone systematic theoretical research to support their practical application [23]. Recently, rotating double prisms have been employed in beam scanners for lidar to achieve wide-range control of multi-beam lasers [24]. Rotating Risley prisms are embedded into an infrared camera to achieve large-scale thermal imaging and monitoring [25]. This means that Risley prisms still have great potential in large-scale and super-resolution imaging.

In this paper, we present a scale-adaptive high-resolution imaging architecture using a rotating-prism-embedded camera. By planning to prism motion, the multi-view images are combined to form a large-scale FOV. If a region of interest (ROI) exists in the combined FOV, the boresight is guided to stare at the ROI to reconstruct the super-resolution (SR) images with the desired information. Our architecture can effectively address the challenge of balancing the requirements of a large FOV and high-resolution imaging, perform distortion and dispersion correction, and alleviate the occlusion to a certain extent. The rest of this paper is summarized as follows. In Section 2, the model of scale-adaptive high-resolution imaging is established based on a rotating-prism-embedded variable-boresight camera. In Section 3, distortion correction, dispersion elimination, and super-resolution imaging are investigated to achieve image enhancement. In Section 4, the experiments are performed to verify the feasibility of our method. Conclusions are drawn in the end.

## 2. The Model of Large-Scale High-Resolution Imaging

**Figure 1:**
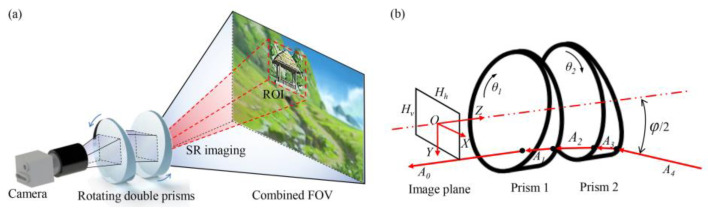
The model of large-scale high-resolution imaging. (**a**) Imaging system. (**b**) Light propagation.

Large-scale high-resolution imaging has wide applications in various scenarios, such as military reconnaissance, security rescue, and scene monitoring. Figure 1a shows the model of large-scale high-resolution imaging. It is composed of a camera to acquire raw images and rotating double prisms for flexible boresight adjustment. By planning the prism motion, the multi-view images are combined to form a large-scale perception FOV for situational awareness. If a region of interest exists in the combined FOV, the boresight is adjusted to stare at the ROI from different viewpoints. As a result, the SR image of the ROI is provided with photo-realistic detailed information by fusing multi-view images. Compared with the traditional multi-camera imaging model, the proposed model can achieve the imaging effect of an infinite number of cameras with just a single camera. The viewpoint adjustment is more flexible, the boresight pointing is more accurate, and the structural configuration is more compact. Figure 1b illustrates the process of light propagation. The coordinate system O-XYZ is established with the optical center of the camera as the origin. According to Snell’s law, we can obtain outgoing lights with *A_1_*=[*H_h_*, *H_v_*, *f*] as the incident light:

(1) $$A_{i+1}=\frac{n_{i}}{n_{i+1}}A_{i}+\left\{\sqrt{1-{\left(\frac{n_{i}}{n_{i+1}}\right)}^{2}\cdot \left[1-{\left(A_{i}\cdot N_{i}\right)}^{2}\right]}-\frac{n_{i}}{n_{i+1}}\left(A_{i,v}\cdot N_{i}\right)\right\}N_{i}={\left[x_{i+1},y_{i+1},z_{i+1}\right]}^{T}$$

where *i* = 0, 1, 2, 3. *n*_1_ and *n*_3_ are the prism refractive index; namely, *n*_1_ = *n*_3_ = *n*. *n*_0_, *n*_2_, and *n*_4_ are the air refractive indices, and namely, *n*_0_ = *n*_2_ = *n*_4_ = 1. *H_h_* and *H_v_* are the sizes of the sensor chip in horizontal and vertical directions. *f* is the focal length of the camera. *N*_1_, *N*_2_, *N*_3_, and *N*_4_ are the normal vectors of the four planes of the prisms:

(2) $$\left\{\begin{array}{l} N_{1}={\left(0,0,1\right)}^{T}\\ N_{2}={\left(\cos\theta_{1}\sin\alpha ,\sin\theta_{1}\sin\alpha ,\cos\alpha \right)}^{T}\\ N_{3}={\left(-\cos\theta_{2}\sin\alpha ,-\sin\theta_{2}\sin\alpha ,\cos\alpha \right)}^{T}\\ N_{4}={\left(0,0,1\right)}^{T} \end{array}\right.$$

where (*θ*_1_, *θ*_2_) represent the rotating angles. α denotes the wedge angle of the prism. Therefore, the FOV angle *φ* of the combined FOV can be deduced as:

(3) $$\varphi =2arc\cos\left(\frac{z_{5}}{\sqrt{x_{5}^{2}+y_{5}^{2}}}\right)$$

## 3. Super-Resolution Imaging with Prism-Induced Distortion Correction

**Figure 2:**
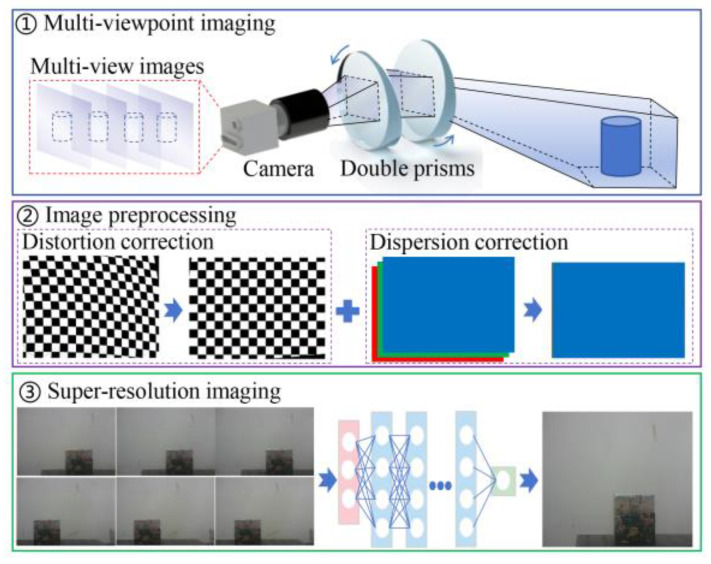
The basic architecture of super-resolution imaging.

Figure 2 shows the basic architecture of rotating-prism-based super-resolution imaging, mainly including multi-view image acquisition using a rotating-prism-embedded variable-boresight camera, multi-view image preprocessing consisting of distortion correction and dispersion correction, and super-resolution imaging by multi-view image fusion. 

As for multi-view imaging based on the variable-boresight camera, the camera boresight is adjusted to capture the multi-view ROI by rotating the double prisms. In terms of multi-viewpoint image preprocessing, it is inevitable that the quality degradation of images will occur, such as distortion and dispersion, because the lights propagate in the non-uniform prisms. Based on the principle that the direction of a light passing through a symmetrical prism remains unchanged, the distortion of multi-viewpoint images is corrected. In addition, the offsets of the three-channel lights are compensated based on Snell’s law to eliminate image dispersion. The deep learning network is utilized to fuse multi-view sequence images to output super-resolution images.

### 3.1. Multi-View Image Preprocessing

#### 3.1.1. Distortion Correction Using Virtual Symmetrical Prisms

**Figure 3:**
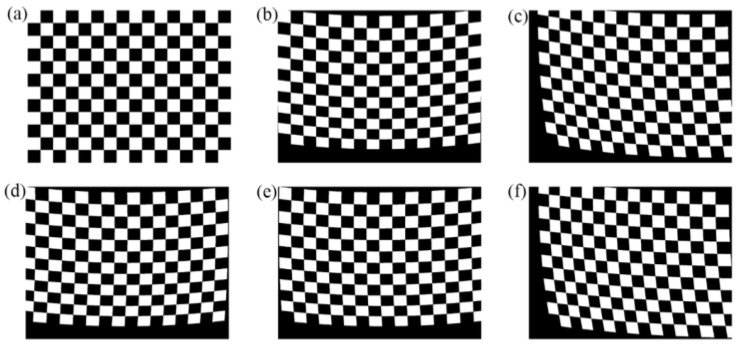
Image distortion under different prism rotation angles. (**a**) Imaging without rotating prisms. (**b**) *θ*_1_ = 0°, *θ*_2_ = 0°. (**c**) *θ*_1_ = 45°, *θ*_2_ = 45°. (**d**) *θ*_1_ = 0°, *θ*_2_ = −45°. (**e**) *θ*_1_ = 0°, *θ*_2_ = 45°. (**f**) *θ*_1_ = 45°, *θ*_2_ = 90°.

The propagation of light in a non-uniform prism does not follow the linear law, which leads to image distortion. Figure 3 shows the image distortion under different prism rotation angles. Each pixel on the camera corresponds to a definite area in the scene, and all pixels form a regular checkerboard image. However, the non-uniform prism has inconsistent refraction capabilities for lights in the FOV, resulting in the originally rectangular checkerboard image no longer being a regular checkerboard image. As the changes to prism rotation angles are undergoing, the distortion of the checkerboard is also constantly changing, including bending deformation, tensile deformation, and compressive deformation.

**Figure 4:**
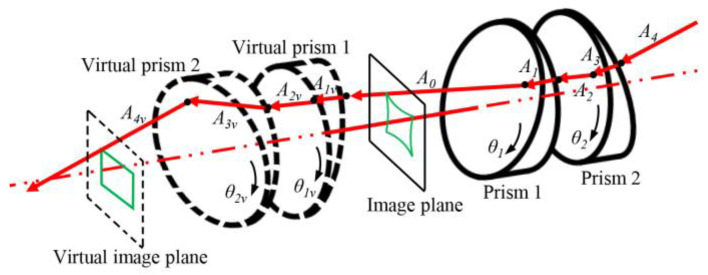
Schematic diagram of the distortion correction by virtual symmetrical prisms.

The directions of the incident and emergent rays are the same when a pair of prisms is symmetrically arranged, and their rotation angles satisfy *θ*_2_ = *θ*_1_ + 180°. Based on this fundamental principle, a pair of virtual symmetrical prisms can be constructed to eliminate the imaging distortion caused by the double prisms as shown in Figure 4.

Two virtual prisms are symmetrically arranged with the image plane as the symmetry plane. The parameters of virtual prisms 1 and 2 are the same as those of real prisms 1 and 2. The rotation angle *θ*_1*v*_ of the virtual prism 1 is *θ*_1_ + 180°, and the rotation angle *θ*_2*v*_ of the virtual prism 2 is *θ*_2_ + 180°. Based on Snell’s law, the incident ray *A*_2_ of prism 1 is in the same direction as the emergent ray *A*_2*v*_ of virtual prism 1. Similarly, the incident ray *A*_4_ of prism 2 is in the same direction as the emergent ray *A*_4*v*_ of virtual prism 2. Furthermore, the distance between the virtual prism 2 and the virtual image plane should be the focal length to obtain the corrected image with the same scale as the original image. To obtain a complete corrected image, each pixel of the distorted image is corrected in sequence. Supposing the image coordinates of a certain point on the distorted image are (*u*, *v*), and the focal length is *f*. So, the incident ray *A*_0_ of virtual prism 1 can be expressed by:

(4) $$A_{0}=\frac{{\left(u,\;v,\;f\right)}^{T}}{\sqrt{\left(u^{2}+v^{2}+f^{2}\right)}}$$

*N*_1,*v*_, *N*_2,*v*_, *N*_3,*v*_, and *N*_4,*v*_ are the normal vectors of the four planes of the virtual prisms:

(5) $$\left\{\begin{array}{l} N_{1,v}={\left(\sin\theta_{1v}\sin\alpha ,-\cos\theta_{1v}\sin\alpha ,\cos\alpha \right)}^{T}\\ N_{2,v}={\left(0,0,1\right)}^{T}\\ N_{3,v}={\left(0,0,1\right)}^{T}\\ N_{4,v}={\left(-\sin\theta_{2v}\sin\alpha ,\cos\theta_{2v}\sin\alpha ,\cos\alpha \right)}^{T} \end{array}\right.$$

According to Equations (1), (4) and (5), we can obtain *A*_1*v*_(*A*_1*vx*_, *A*_1*vy*_, *A*_1*vz*_), *A*_2*v*_(*A*_2*vx*_, *A*_2*vy*_, *A*_2*vz*_), *A*_3*v*_(*A*_3*vx*_, *A*_3*vy*_, *A*_3*vz*_), and *A*_4*v*_(*A*_4*vx*_, *A*_4*vy*_, *A*_4*vz*_). Any light passing through the virtual double prisms can be determined by its direction vector and a point on the light:

(6) $$\frac{x-x_{p}}{A_{ivx}}=\frac{y-y_{p}}{A_{ivy}}=\frac{z-z_{p}}{A_{ivz}},i=1,2,3,4$$

where (*x_p_*, *y_p_*, *z_p_*) is the point on the light. The virtual imaging plane and planes of the virtual prisms can be expressed by:

(7) $$\left(x-x_{b}\right)N_{x}-\left(y-y_{b}\right)N_{y}+\left(z-z_{b}\right)N_{z}=0$$

where [*N_x_*, *N_y_*, *N_z_*] is the normal vector of a plane. (*x_b_*, *y_b_*, *z_b_*) is a point on the plane. Combining Equations (6) and (7), we can obtain the intersection between the virtual imaging plane and the light determined by *A*_4*v*_, namely corrected image coordinates *P_v_* = (*x_v_*, *y_v_*, *f*)^T^. The pixel coordinates corresponding to *P_v_* are as follows:

(8) $$\left(u_{v},v_{v}\right)=\left(\frac{x_{v}}{d_{x}}+u_{0},\frac{y_{v}}{d_{y}}+v_{0}\right)$$

where *d_x_* and *d_y_* are the physical dimensions of pixels in the horizontal and vertical directions. (*u*_0_, *v*_0_) is the principal point of the camera.

#### 3.1.2. Dispersion Elimination Based on Reverse Tracing

**Figure 5:**
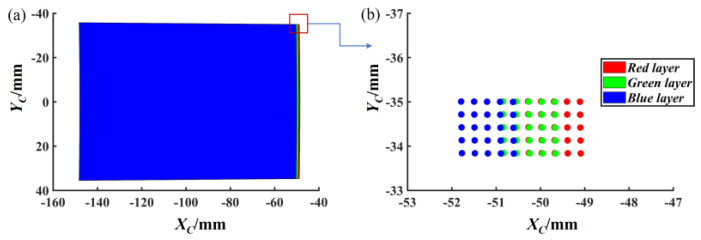
Schematic diagram of image dispersion. (**a**) Overall dispersion effect. (**b**) Magnified local view.

Image dispersion can lead to reduced contrast and color distortion, all of which directly reduce the visual effect of images. During the imaging process of an RGB camera, the three basic colors (red, blue, and green) are mixed in a certain proportion to form lights of any color. The lights with different colors have different wavelengths; namely, their refractive indices are different. Therefore, the camera combined with the double prisms will cause the image dispersion as shown in Figure 5.

As shown in Figure 5a, pixel-level offsets occur on the three-channel layers because the wavelengths of the red, green, and blue lights provide the different refractive indices in the prisms. Figure 5b shows the 5 × 5-pixel area in the upper right corner of the imaging plane. Because the refractive index of the wavelength of blue light is the largest, the offset of the blue channel in the image plane is the largest. This means that the three-channel offsets on the image plane can be determined under the condition that the parameters of the imaging system are known. Therefore, these offsets can be compensated to eliminate dispersion. According to Section 3.1, the pixel coordinates of each channel after distortion correction can be obtained:

(9) $$\left\{\begin{array}{l} p_{vr}={\left(u_{vr},v_{vr}\right)}^{T}\\ p_{vg}={\left(u_{vg},v_{vg}\right)}^{T}\\ p_{vb}={\left(u_{vb},v_{vb}\right)}^{T} \end{array}\right.$$

Taking the red channel as the reference, the dispersion elimination adjustment amounts for the green and blue channels are:

(10) $$\left\{\begin{array}{l} \Delta p_{vg}=\left|p_{vg}-p_{vr}\right|\\ \Delta p_{vb}=\left|p_{vb}-p_{vr}\right| \end{array}\right.$$

### 3.2. Super-Resolution Imaging by Multi-Viewpoint Image Fusion

**Figure 6:**
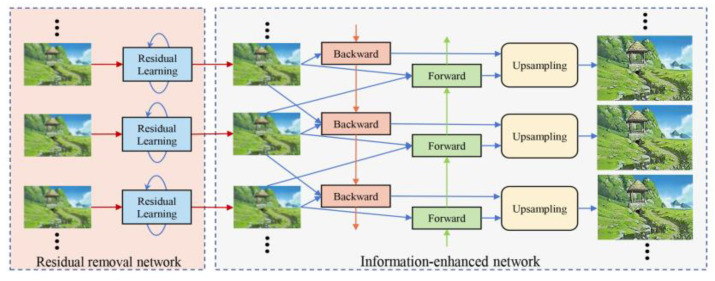
The basic schematic diagram of super-resolution imaging by multi-view image fusion.

Figure 6 shows the basic schematic diagram of super-resolution imaging by multi-view image fusion. The imaging system adjusts the prism rotation angle to capture multi-frame sequence images from different perspectives. The acquired multi-view images are independently input to the residual removal network for degradation removal. Subsequently, the clean image without artifacts enters the information enhancement network to output the super-resolution images.

**Figure 7:**
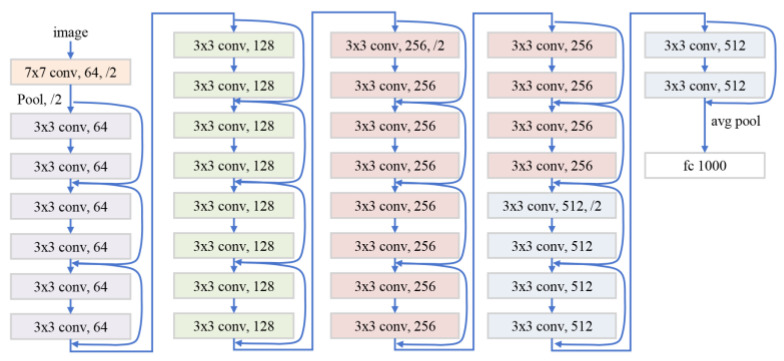
Network architecture for the residual block.

Since there are inevitably some noises in the image, such as artifacts, and residual distortions, these image degradations need to be removed before they are input into the network. Let *x_i_* be the captured raw image from the *i*-th viewpoint, and *R* be the residual removal network that is 20 residual blocks, that is:

(11) $${\tilde{x}}_{i}=R(x_{i})$$

where ${\tilde{x}}_{i}$ are the images with degradation removal. The network architecture for the residual block is shown in Figure 7.

**Figure 8:**
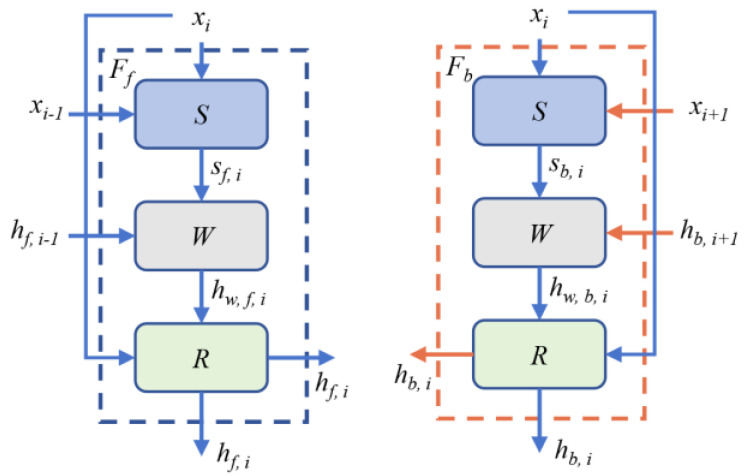
Network architecture for the forward propagation and backward propagation.

Then, the clean sequence images are passed to the information enhancement network consisting of backward propagations, forward propagations, and upsampling modules. The network architectures for the backward propagation and forward propagation are shown in Figure 8. Specifically, the neighboring frame images of *x_i_* are *x_i−1_* and *x_i+1_*. In addition, the corresponding features from neighboring images are *h_f, i−1_* and *h_f, i+1_*. We can obtain:

(12) $$\left\{\begin{array}{l} h_{f,i}=F_{f}\left(x_{i,}x_{i-1,}h_{f,i-1}\right)\\ h_{b,i}=F_{b}\left(x_{i,}x_{i+1,}h_{b,i-1}\right) \end{array}\right.$$

where *F_b_* is the backward propagation, and *F_f_* is the forward propagation. The backward and forward propagations include the flow estimation module *W* [26], the spatial warping module *S* [27], and the residual blocks *R*. The propagation process of the backward and forward propagations can be expressed by:

(13) $$\left\{\begin{array}{l} s_{b/f,\;i}=S\left(x_{i},\;x_{i\pm 1}\right)\\ h_{w,\;b/f,\;i}=W\left(h_{w,\;b/f,\;i\pm 1},s_{b/f,\;i}\right)\\ h_{b/f,\;i}=R\left(x_{i,}\;h_{w,\;b/f,\;i}\right) \end{array}\right.$$

The upsampling module consists of multiple convolutions and pixel-shuffle [28]. Here, it is named *V*:

(14) $$\left\{y_{i}\right\}=V\left(\left\{{\tilde{x}}_{i}\right\}\right)$$

A low-resolution true value is used to constrain the output of the residual removal network:

(15) $$\eta =\sum_{i=1}^{n}\delta ({\tilde{x}}_{i}-m(b_{i}))$$

where *b_i_* represents the true value of the original high-resolution image, *m* represents the downsampling operator, and δ represents Charbonnier loss function. In many cases, introducing the residual removal network only once cannot effectively eliminate excessive image degradation. The repeated introduction of the residual removal network is prone to causing image distortion. For this reason, a dynamic optimization scheme is proposed:

(16) $$\left\{\begin{array}{l} {{\tilde{x}}_{i}}^{j+1}=R({{\tilde{x}}_{i}}^{j})\;\;\;\;\;\;\;if\;\sum_{j=1}^{n}\left|{{\tilde{x}}_{i}}^{j}-{{\tilde{x}}_{i}}^{j-1}\right|/n\geq \sigma ,\;\\ {\tilde{x}}_{i}={{\tilde{x}}_{i}}^{j}\;\;\;\;\;\;\;\;\;\;\;\;\;\;otherwise \end{array}\right.$$

where *σ* is a pre-determined stop threshold. After conducting multiple tests, it is determined that setting σ to 1.5 is appropriate.

As for architecture, a convolution is employed to capture the shallow features in the image. Additionally, the deep features in the image are extracted by 20 residual blocks. Finally, the convolution layer is used to generate clean images. In term of training settings, the REDS dataset [29] is employed to train this network. Adam optimizer [30] is adopted with constant learning rates. The patch size of the input low-resolution image is set to 64 × 64. In the process of the training, the pre-train is performed with output loss and cleaning loss. Specifically, the number of iterations is 300 K, the batch size is 16, and the learning rate is 10^−4^. Then, the network is finetuned with perceptual loss [31]. Specifically, the number of iterations is 150 K, the batch size is 8, and the learning rate is 5 × 10^−5^.

## 4. Experiment

### 4.1. Simulation Experiment

To further demonstrate the superiority of the system, simulation experiments were performed in expanding FOV and boresight pointing ranges. The parameters of the simulation system are as follows: *α* = 10°, *n* = 1.517, the thin end thickness of the prism is *D*_0_ = 3 mm, spacing between the double prisms is *D* = 3 mm, and the sampling plane distance from the double prisms is 500 mm.

**Figure 9:**
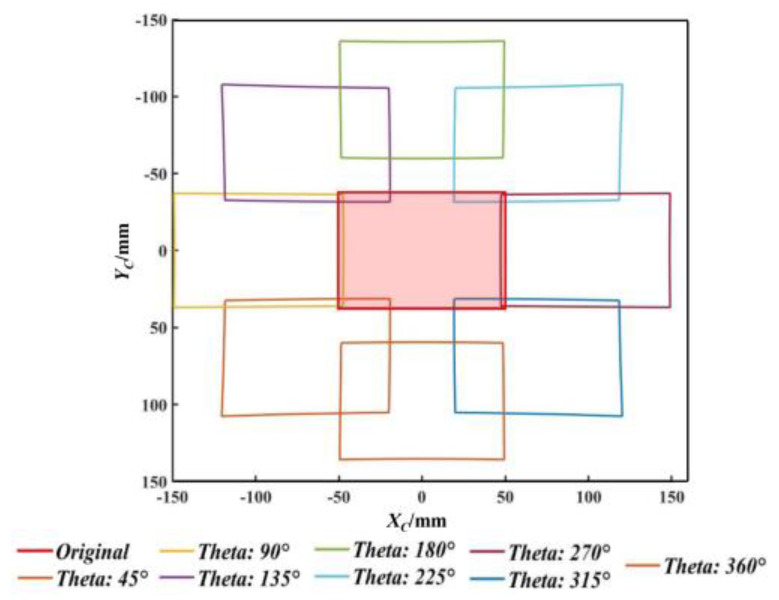
The imaging FOV in different rotation angles.

The pitch angle of boresight is the largest when the rotation angles of the two prisms in the double prisms are the same, that is, the deflection effect on the imaging light is the most significant. Therefore, by driving the rotation angles to be the same and taking 45° as the step size, the imaging range of the system is displayed as shown in Figure 9. The red area in the figure represents the original FOV of the camera. Specifically, the angle of horizontal FOV *φ_H_*_0_ is 8.84°, while the angle of vertical FOV *φ_V_*_0_ is 6.64°. The horizontal combined FOV can obtain its extreme value when the rotation angles reach 90° and 270°, respectively. Similarly, their vertical combined FOV reaches its extreme value when the rotation angles are 0° and 180°. According to Equation (3), it is calculated that the horizontal and vertical combined FOVs are 26° and 23.67°. The combined FOV has been improved by approximately three times compared to the camera’s original FOV.

In order to deeply analyze the effect of system parameters on the imaging range expansion, the control variable method is adopted for quantitative analysis. The key analysis focuses on the variation trends of the horizontal combined FOV *φ_H_* and the vertical combined FOV *φ_V_* with *α* and *n*. To directly demonstrate the capabilities of these two parameters on the FOV expansion, the horizontal and vertical FOV magnification are defined as *K_H_* = *φ_H_*/*φ_H_*_0_ and *K_V_* = *φ_V_*/*φ_V_*_0_.

**Figure 10:**
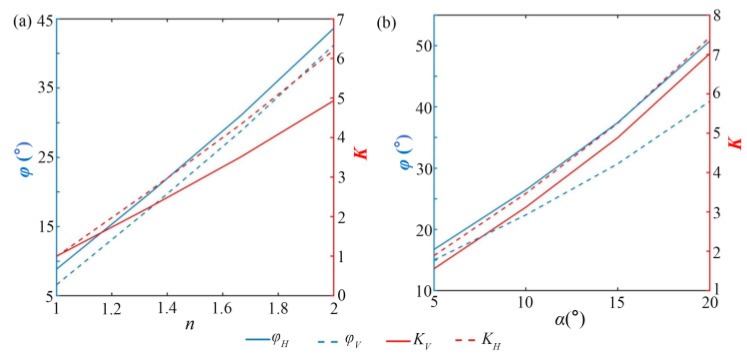
The variation law of FOV. (**a**) The variation of FOV with the increase in *α*. (**b**) The variation of FOV with the increase in *n*.

Figure 10 shows the variation laws of *n* and *α* on the FOV expansion. It can be seen from Figure 10a that the physical properties of the prism are similar to those of air when the refractive index approaches 1, that is, the imaging light passing through the prism hardly undergoes refraction. Meanwhile, *K_H_* and *K_V_* approach 1; namely, the effect of expanding the FOV is not obvious. However, *φ_H_*, *φ_V_*, *K_H_*, and *K_V_* keep increasing with the increase of *n*, roughly showing a linear relationship. Similarly, *φ_H_* and *φ_V_* keep expanding as *α* increases as shown in Figure 10b. *K_H_* and *K_V_* also increase with the increase of *α*, that is, the FOV expansion ability is stronger. Compared with *n*, *α* has a more significant effect on expanding FOV. The reason for this variation rule is that the refraction level of light in a prism has a nonlinear relationship with *α*.

**Figure 11:**
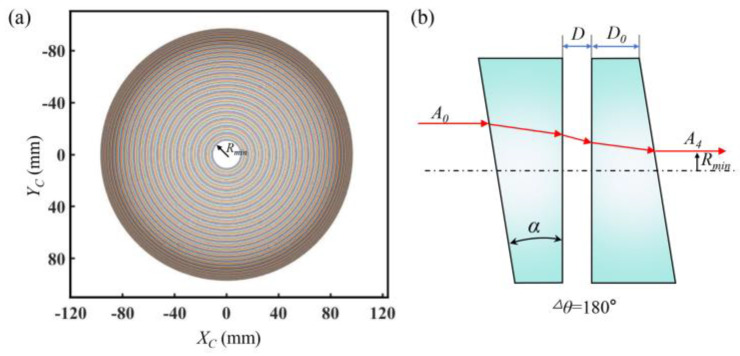
Schematic diagram of the scanning region. (**a**) Scanning region. (**b**) Causes of the blind area.

Figure 11a shows the boresight pointing area on the sampling plane. There is a maximum pointing boundary when the double prisms with a 0° angle difference rotate synchronously. In addition, the refraction effects of the two prisms on the lights cancel each other out when their angle difference is 180°. As a result, the emergent light vector *A*_4_ and the incident light vector *A*_0_ are in the same direction and both along the optical axis. However, there is a certain gap *D* between the two prisms due to the system installation requirements as shown in Figure 11b. Therefore, there must be a certain area on the sampling plane that the boresight cannot reach, namely the pointing blind zone. Specifically, the radius of the pointing blind zone is represented by *R_min_*. The influences of *n*, *α*, and *D* on the pointing blind zone were analyzed.

**Figure 12:**
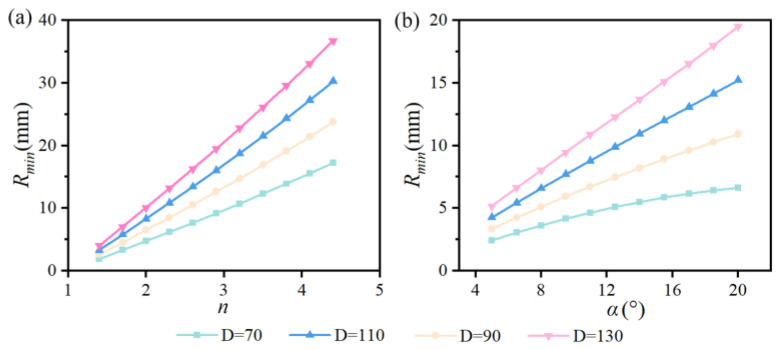
Blind zone distribution. (**a**) The influence of α and *D* on the blind zone. (**b**) The influence of *n* and *D* on the blind zone.

Figure 12 shows the blind zone distribution under different *α*, *n,* and *D*. It can be seen from Figure 12a that *R_min_* gradually increases with an increase in *n* when *D* does not change. In addition, *R_min_* also increases along with the increase of *D* under the condition that *n* is constant. Similarly, *R_min_* also keeps increasing with the increase of *α* when *D* is a constant value as shown in Figure 12b. *R_min_* also gradually increases with an increase in *D* given that *α* remains constant. In particular, the degree of blind zone expansion is becoming increasingly significant at a high level of constant *D*. By further comparing Figure 10a, Figure 10b, Figure 12a and Figure 12b comprehensively, we can conclude that increasing *α* and *n* can effectively expand the imaging range, but it will also expand the blind area. Therefore, the gap between the double prisms must be minimized as much as possible to reduce the blind zone expansion caused by the increase in *α* and *n* for expanding FOV.

### 4.2. Real Experiment

**Figure 13:**
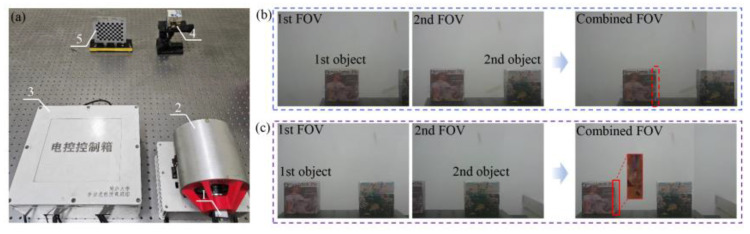
Experimental setup and scene perception. (**a**) Experimental setup. (**b**) FOV expansion. (**c**) Viewpoint adjustment.

Figure 13a shows the experimental setup, mainly including (1) a camera, (2) rotating double prisms, (3) a control system, (4) an object, and (5) a calibration board. The rotating double prisms are coaxially arranged with the camera, and the double prisms can be rotated to adjust the boresight pointing arbitrarily. The control system is capable of enabling the double prism to rotate freely within a 360° range. The objects to be imaged are set within the combined FOV of the camera and the double prisms. The camera captures multi-view images of the calibration board to calibrate the parameters of the camera based on the previous work [32]. Specifically, the parameters of the camera and the double prisms are shown in Table 1.

To verify the large-FOV imaging feasibility of the proposed architecture, large-scale imaging experiments were carried out. The first object and the second object were imaged and combined from different perspectives as shown in Figure 13b. Usually, we want to obtain more feature information to assist in object recognition. However, no more information from the side of the target can be obtained from a fixed viewpoint due to the object self-occlusion as shown in the red dashed box in Figure 13c. It is notable that the occlusion is overcome by viewpoint adjustment as shown in the red solid line box in Figure 13c; namely, the rich information from the side of the target can be obtained.

**Figure 14:**
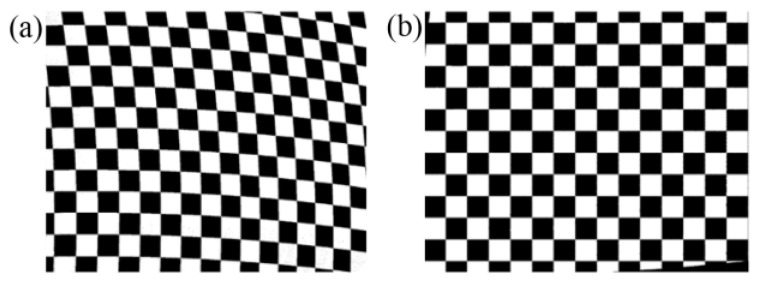
Correction image comparison. (**a**) Distortion image. (**b**) Correction image.

To demonstrate the feasibility of the proposed distortion correction method, a distortion correction experiment was carried out. The calibration board was captured by the camera embedded in the rotating double prisms as shown in Figure 14a. We can see that there is an obvious distortion in the calibration board image. The regular black and white rectangles have turned into rhombuses. The horizontal and the vertical lines have turned into curves. The distortion correction method proposed in Section 3.1 was adopted to correct the distorted image of the calibration board, of which the results are shown in Figure 14b. We can see that the black and white rhombuses have been corrected to regular rectangles, and the horizontal and vertical lines have basically been restored to straight lines. This means that our method can correct the distortion caused by the nonlinear propagation of light in uneven prisms.

**Figure 15:**
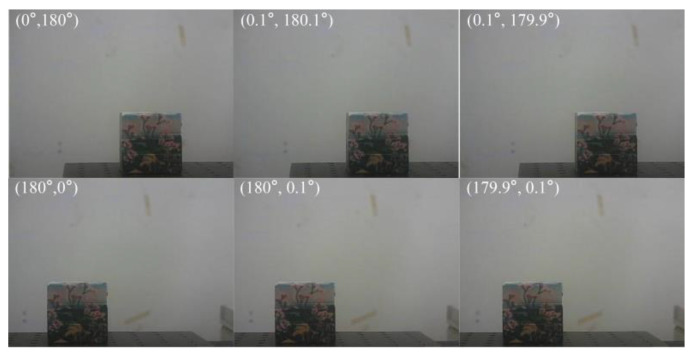
Multi-viewpoint image sequence.

Another important experiment is the SR imaging of the object of interest. The object of interest was captured under different rotation angle combinations of prisms, such as (0°, 180°), (0.1°, 180.1°), (0.1°, 179.9°), (180°, 0°), (180.1°, 0.1°), and (179.9°, 0.1°). The collected multi-viewpoint images are shown in Figure 15.

**Figure 16:**
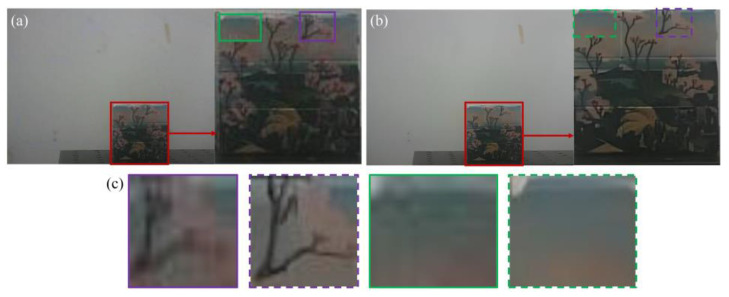
Image comparison. (**a**) Original image. (**b**) SR image. (**c**) Image comparison.

The collected sequence images are preprocessed by distortion correction and dispersion elimination. Subsequently, the corrected images were input into the proposed super-resolution model to output the SR image as shown in Figure 16. The resolution magnification factor *K* = 4, that is, the image resolution is (320 × *K*) × (240 × *K*) = 1280 × 960.

It can be clearly seen in Figure 16a,b that the details and textures in the SR image have been significantly improved compared with the original image. More texture and feature information can be identified in the SR image. Specifically, the aliased green and black backgrounds in the original image are restored and distinguishable in the SR image. In addition, the branches in the purple box become recognizable in the SR image, while the branches are blurred or even unrecognizable in the original image. In particular, the artifacts and dispersion in the SR image are significantly eliminated compared with the original image as shown in the green box in Figure 16c.

To quantitatively analyze the optimization effect of this method on image clarity and texture features, a comparative verification experiment was carried out. The comparison results are shown in Table 2. Nearest Super-Resolution (Nearest SR) [33], Bilinear Super-Resolution (Bilinear SR) [34], and Bicubic Super-Resolution (Bicubic SR) [35] methods were adopted to perform super-resolution imaging on the above-mentioned original images at the same magnification, respectively. Quantitative analysis was conducted based on the three common evaluation indicators for image clarity, including the Brenner gradient, Tenengrad gradient, and discrete cosine transform (DCT). Bilinear SR and Bicubic SR have similar imaging quality in the three evaluation indicators, but the imaging quality of the two methods is the poorest, followed by Nearest SR. Our method outperforms other methods in all three evaluation indicators and particularly stands out in the Brenner gradient. In addition, the proposed method is compared with the two methods (BSRGAN [10] and Real-ESRGAN [36]) based on deep learning. NIQE [37] and BRISQUE [38] are used for qualitative comparison. The comparison results are shown in Table 3. The imaging quality of BSRGAN and Real-ESRGAN is similar in terms of NIQE and BRISQUE. However, the proposed method outperforms the other two methods in both of these metrics, especially in BRISQUE.

## 5. Conclusions

In this paper, we present a scale-adaptive high-resolution imaging architecture using a rotating-prism-embedded camera. By planning to prism motion, the multi-view images are combined to form a large-scale FOV. If a region of interest (ROI) exists in the combined FOV, the boresight is guided to capture the ROI from different viewpoints to get the super-resolution (SR) image with the desired information. A novel distortion correction method is proposed using virtual symmetrical prisms with rotating supplementary angles, which can eliminate image distortion caused by non-uniform refraction. Based on light reverse tracing, the dispersion induced by monochromatic lights with different refractive indices passing through prisms can be eliminated by accurate pixel-level position compensation. For resolution enhancement, we provide a new scheme for SR imaging consisting of the residual removal network for artifact removal and the information enhancement network to improve resolution by multi-view image fusion. The influence of system parameters on expanding FOV and boresight pointing ranges was analyzed to guide the system design. The experiments show that the proposed architecture can achieve both large-FOV scene imaging for situational awareness and SR ROI display to acquire details, effectively perform distortion and dispersion correction, and alleviate occlusion to some extent. It also provides higher image clarity compared to the traditional SR methods. However, the proposed method still has certain limitations. The process of multi-viewpoint image acquisition and distortion correction for large-scale imaging still requires a certain amount of time. In the future, we will continue to explore strategies for enhancing the efficiency of this method, such as algorithm lightweighting, increases in system rotation speeds, and improvement of imaging frame rates.

## Figures and Tables

**Table 1:** System parameters.

Prism Diameter (mm)	*D*_0_ (mm)	*n*	*α* (°)	Camera Internal Parameters	Distortion Coefficient
80	5	1.517	10	[7321.2, 0, 0; 0, 7427.4, 0; 579.1, 562.5, 1]	(−0.67, 8.26)

**Table 2:** Comparison of the interpolation-based Methods.

Method	Nearest SR	Bilinear SR	Bicubic SR	Proposed method
Brenner (∙10^9^)	1.03	0.30	0.45	1.31
Tenengrad (∙10^7^)	5.16	2.03	2.72	6.20
DCT (∙10^6^)	1.99	1.39	1.49	2.24

**Table 3:** Comparison of the methods based on deep learning.

Method	BSRGAN	Real-ESRGAN	Proposed Method
NIQE	5.43	5.39	4.45
BRISQUE	34.71	35.12	31.24

## Data Availability

The original contributions presented in this study are included in the article. Further inquiries can be directed to the corresponding authors.
